# Corrigendum to: Next generation sequencing applied to food safety. A new professional profile

**DOI:** 10.1093/eurpub/ckab184

**Published:** 2021-12-31

European Journal of Public Health, 2020; https://dx.doi.org/10.1093/eurpub/ckaa166.682

In the originally published version of this abstract, the name and affiliation of author F. Pompei were incorrectly given as *S. Pompei*, *Istituto Zooprofilattico Sperimentale dell’Abruzzo e del Mol, Regione Abruzzo-Molise, Teramo, Italy*.

This has now been corrected to *F. Pompei, Istituto Zooprofilattico Sperimentale dell' Abruzzo e del Molise “G.Caporale” Teramo, Italy*.

